# The Skin-Bone Axis in Psoriasis: An Emerging Osteoimmunological Perspective

**DOI:** 10.3390/jcm15208066

**Published:** 2026-10-17

**Authors:** Natsuko Saito-Sasaki, Yu Sawada

**Affiliations:** Department of Dermatology, University of Occupational and Environmental Health, 1-1 Iseigaoka, Yahatanishi-ku, Kitakyushu 807-8555, Fukuoka, Japan

**Keywords:** psoriasis, osteoimmunology, bone metabolism, RANKL–RANK–OPG axis, IL-17/IL-23 axis, bone marrow immune niche, skin–bone axis

## Abstract

Psoriasis is a chronic inflammatory skin disease accompanied by systemic comorbidities such as metabolic syndrome and cardiovascular disease. Bone homeostasis is tightly regulated by immune cells through the receptor activator of nuclear factor-κB ligand (RANKL)–RANK–osteoprotegerin (OPG) axis and inflammatory cytokines including tumor necrosis factor-α (TNF-α) and interleukin (IL)-17. Although bone destruction has been extensively characterized in psoriatic arthritis (PsA), whether the skeleton is affected in psoriasis without clinically apparent arthritis remains unsettled: population-based data suggest a modest, inconsistently replicated excess of fractures, yet pooled analyses show no corresponding reduction in areal bone mineral density. This review integrates evidence from osteoimmunology, the bone marrow immune niche, microbiome research, and clinical studies to propose the skin–bone axis as a conceptual framework linking chronic cutaneous inflammation to systemic skeletal remodeling beyond joint involvement. Because the strength of the supporting evidence varies widely across these domains, we grade each component of the proposed axis explicitly as established, extrapolated, or hypothetical, identifying bone marrow niche remodeling, trained immunity, and microbiome-mediated skeletal regulation as hypothesis-generating rather than demonstrated, and we appraise the confounding introduced by lifestyle, comorbidity, and cumulative corticosteroid exposure. We conclude by defining the knowledge gaps and study designs required to determine whether bone health should be incorporated into psoriasis management.

## 1. Introduction

Psoriasis is a chronic immune-mediated inflammatory skin disease that affects approximately 2–3% of the global population. Beyond cutaneous manifestations, psoriasis is now firmly established as a systemic inflammatory disorder [1], with consistent associations with dyslipidemia [2], cardiovascular disease (increased risks of coronary artery disease, stroke, and cardiovascular mortality) [3], inflammatory bowel disease [4], non-alcoholic fatty liver disease, chronic kidney disease, psychiatric symptoms including depression and anxiety, and psoriatic arthritis (PsA) [1]. The systemic dimension is particularly evident in severe forms such as generalized pustular psoriasis, in which multi-organ inflammatory dysfunction including acute respiratory failure, hepatic and renal dysfunction, and cardiovascular complications is increasingly recognized [5]. Psoriatic lesions show metabolic reprogramming of infiltrating immune cells and keratinocytes, including enhanced glycolysis, mTOR activation, and altered lipid metabolism, which sustains chronic inflammation and tissue remodeling [6]. Consistently, single-cell transcriptomic analyses demonstrated that immunometabolic pathways are closely associated with inflammatory reprogramming in circulating CD4^+^ central memory and CD8^+^ effector memory T cells in psoriasis and PsA, while activation of these pathways may concurrently enhance regulatory T cell (Treg)-mediated immune suppression, suggesting nutrient and lipid metabolism as candidate therapeutic targets [7]. These findings further support the concept of psoriasis as a systemic immune-metabolic disease.

Bone tissue is maintained through tight interaction with the immune system. The receptor activator of nuclear factor-κB ligand (RANKL)–RANK–osteoprotegerin (OPG) axis is the central regulator of osteoclast differentiation [8]. The inflammatory cytokines enhance RANKL signaling and osteoclastogenesis, whereas interferons and OPG suppress this process [9]. This bone–immune crosstalk has been systematized as osteoimmunology and has been most extensively characterized in rheumatoid arthritis [10].

In PsA, enhanced osteoclast differentiation mediated by tumor necrosis factor-α (TNF-α) and RANKL drives bone erosion [11]. Accordingly, bone metabolic abnormalities in psoriatic disease have largely been investigated in the context of joint involvement. However, in patients with psoriasis not selected for joint disease, elevations of bone- and cartilage-related serum biomarkers such as cartilage oligomeric matrix protein (COMP), OPG, and interleukin (IL)-20 [12], reductions in lumbar spine and femoral neck bone mineral density (BMD) in small cross-sectional studies [13,14], one of which included patients with PsA [14]—a finding not confirmed in a subsequent meta-analysis [15]—and a modestly increased risk of fracture in some, but not all, population-based cohorts [16,17] have been reported. In most of these studies, however, PsA was not systematically excluded, so that the findings cannot yet be attributed to cutaneous inflammation alone. Despite this accumulating evidence, a coherent conceptual framework integrating these observations has not yet been established. This review provides an integrative overview of the relationship between cutaneous inflammation and bone metabolism in psoriasis from the perspectives of osteoimmunology, the bone marrow niche, mechanistic and clinical–therapeutic data, and proposes a framework within which skin-driven skeletal effects, independent of joint inflammation, can be tested.

This is a narrative review. The literature was identified through iterative searches of PubMed (MEDLINE) from database inception to March 2026, restricted to English-language publications, using combinations of terms relating to psoriasis and psoriatic arthritis with terms relating to bone mineral density, osteoporosis, fracture, osteoimmunology, the bone marrow niche, trained immunity and the microbiome; no restriction by publication type was applied. Because this is a narrative review, a single predefined search string was not used; the database, search period, eligibility criteria and the key terms and their combinations are provided in Supplementary Table S1. The reference lists of retrieved articles were hand-searched, and targeted searches for recently published studies were repeated during manuscript revision (September 2026). Literature screening, data extraction and the assignment of evidence levels were performed by one author (N.S.-S.); dual independent screening was not undertaken, so that no procedure for resolving disagreements between reviewers was required. Because a principal aim of this review is to separate what has been demonstrated from what is at present only plausible, each body of evidence was appraised against an explicit three-tier scheme, applied to the claim rather than to the individual study (Table 1). Evidence was graded as established (Level A) when consistent human data are available directly in psoriatic disease, from more than one independent cohort or from meta-analysis, or when a mechanism has been demonstrated in psoriatic tissue; as extrapolated (Level B) when the supporting data are robust but derive from other chronic inflammatory diseases, from animal models of skin inflammation, or from general bone biology, and therefore require an inferential step to reach human psoriasis; and as hypothetical (Level C) when a mechanism is biologically plausible but no direct supporting data in psoriasis exist, so that the proposition is hypothesis-generating only. This scheme is an operational construct rather than a validated instrument such as GRADE, and it is used here to make the basis of each statement explicit rather than to quantify certainty. Statements resting on Level B or Level C evidence are identified as such throughout, and Section 5 and Section 6 are, in their entirety, Level C.

Because the central question of this review concerns skeletal involvement in psoriasis without arthritis, the population from which each clinical finding derives is specified throughout using five categories: (i) cutaneous psoriasis with PsA prospectively excluded by rheumatologic assessment; (ii) cutaneous psoriasis without systematic exclusion of PsA; (iii) mixed psoriasis/PsA populations; (iv) PsA alone; and (v) non-psoriatic inflammatory diseases or experimental models. The category of the principal supporting sources is shown for each component of the proposed axis in Table 1. Screening questionnaires were not regarded as equivalent to rheumatologic assessment, because they cannot reliably exclude subclinical enthesitis or synovitis. Throughout, “psoriatic disease” refers to cutaneous psoriasis and PsA collectively, “cutaneous psoriasis” to patients without a diagnosis of PsA, and “PsA” to patients with psoriatic arthritis.

## 2. Psoriasis Pathophysiology

Psoriasis is a chronic, immune-mediated inflammatory disease affecting approximately 2–3% of the population and arising from the interplay between genetic susceptibility and environmental triggers. The strongest genetic determinant is the HLA-C*06:02 risk allele, and common triggers include streptococcal infection, mechanical trauma (the Koebner phenomenon), smoking, obesity, and psychological stress [30]. Over the past two decades, the pathogenesis of psoriasis has been reconceptualized around the IL-23/IL-17 immune axis [31]. Following an initial trigger, dendritic cells are activated—in part by the antimicrobial peptide LL-37 complexed with self-nucleic acids—and produce IL-23 and IL-12. IL-23 in particular sustains and expands a heterogeneous population of IL-17–producing cells, including T helper 17 (Th17) cells, cytotoxic Tc17 cells, γδ T cells, and group 3 innate lymphoid cells [32].

These cells secrete IL-17A, IL-17F, IL-22, and TNF-α, which act on epidermal keratinocytes to induce hyperproliferation, incomplete differentiation, and the release of antimicrobial peptides, chemokines, and additional proinflammatory cytokines [31]. The activated keratinocytes thereby amplify the inflammatory signal, establishing a self-sustaining “feed-forward” loop that recruits neutrophils and inflammatory myeloid cells and maintains the mature psoriatic plaque. The pathogenic primacy of this axis is demonstrated by the marked clinical efficacy of biologic agents that neutralize TNF-α, IL-17A, or IL-23 in achieving and maintaining skin clearance [30]. This therapeutic evidence, together with genetic and transcriptomic data, has firmly established psoriasis as a T cell– and cytokine-driven disease rather than a primary disorder of keratinocyte proliferation.

Importantly, the biological effects of the IL-23/IL-17 axis are not confined to the skin. IL-17A acts on a broad range of mesenchymal and stromal targets, including endothelial cells, fibroblasts, chondrocytes, osteoblasts, and osteoclasts, thereby coupling cutaneous inflammation to remodeling in distant tissues [33]. In parallel, psoriatic inflammation is accompanied by systemic immunometabolic reprogramming of circulating immune cells that reinforces the chronic inflammatory state [6,7]. These features underlie the now well-established concept of psoriasis as a systemic inflammatory disease associated with multiple comorbidities beyond the skin [1]. Because the cytokines that drive cutaneous disease also regulate bone-resident and immune cells, the immunologic circuitry of psoriasis provides a plausible mechanistic entry point for the skeletal effects that are the focus of this review.

## 3. Osteoimmunology Overview

**Figure 1:**
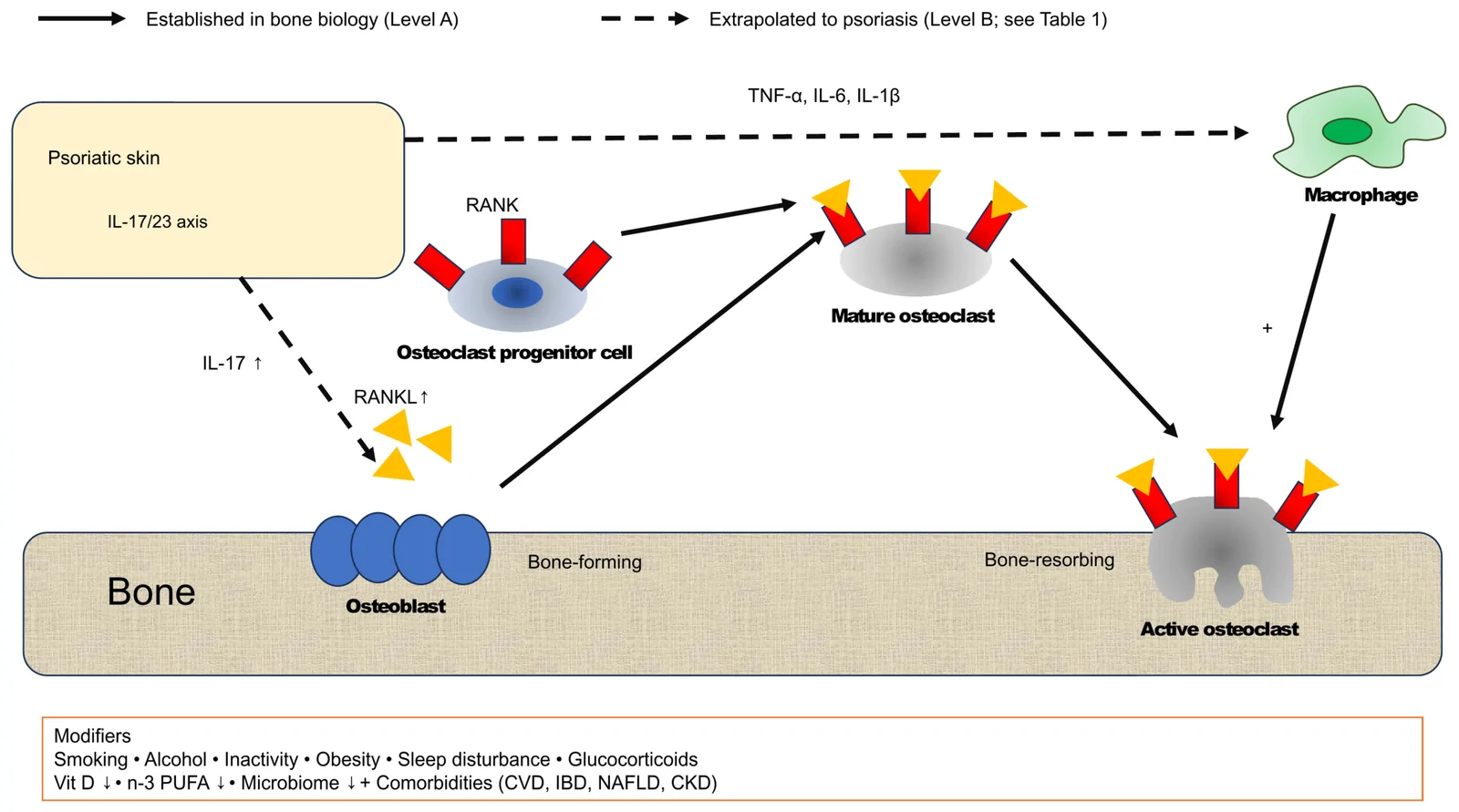
Proposed osteoimmunological mechanisms linking cutaneous inflammation to bone remodeling in psoriasis. In psoriatic skin, activation of the IL-23/IL-17 axis generates a systemic inflammatory milieu. IL-17 upregulates RANKL on osteoblasts and mesenchymal stromal cells; RANKL engages RANK on osteoclast progenitor cells and drives their differentiation into mature and subsequently bone-resorbing osteoclasts, shifting the remodeling balance away from osteoblast-mediated bone formation. Circulating TNF-α, IL-6 and IL-1β additionally recruit and activate macrophages, which serve both as osteoclast precursors and as a further source of osteoclastogenic cytokines. Lifestyle factors, medication exposure and comorbidities (lower box) act as modifiers of bone metabolism and as potential confounders of the epidemiologic associations discussed in Section 4.4. Solid arrows denote osteoimmunological steps established in bone biology (Level A as bone biology); dashed arrows denote links from cutaneous inflammation to bone and immune cells that are extrapolated to psoriasis from other inflammatory diseases and animal models (Level B; Table 1). Upward and downward arrows (↑, ↓) indicate increased and decreased levels, respectively, and “+” indicates a promoting effect. The pathways depicted correspond to those graded Level A or B in Table 1; the bone marrow niche and microbiome–bone mechanisms discussed in Section 5 and Section 6 are hypothetical and are therefore not depicted as pathways, the gut microbiome appearing only in the lower panel among the modifiers. CKD, chronic kidney disease; CVD, cardiovascular disease; IBD, inflammatory bowel disease; IL, interleukin; NAFLD, non-alcoholic fatty liver disease; PUFA, polyunsaturated fatty acid; RANK, receptor activator of nuclear factor-κB; RANKL, RANK ligand; TNF-α, tumor necrosis factor-α; Vit D, vitamin D.

Bone remodeling reflects the coordinated activity of osteoclast-mediated bone resorption and osteoblast-driven bone formation [34]. Inflammatory cytokines such as TNF-α, IL-1β, IL-6, and interferons can disrupt this coupling, leading to pathological bone loss or ectopic bone formation [35] (Figure 1). IL-17A produced by Th17 cells and other IL-17-producing populations (including Tc17 cells, γδ T cells, and group 3 innate lymphoid cells) [33] upregulates RANKL expression in osteoblasts, synovial fibroblasts, and mesenchymal stromal cells, while concurrently upregulating RANK on osteoclast precursors. Together with TNF-α and IL-6, this synergistically promotes osteoclast differentiation and tilts the remodeling balance toward bone resorption [18,19]. In contrast, Tregs suppress osteoclastogenesis through OPG induction and cell-contact–dependent inhibition of RANKL signaling; type I interferons (IFN-α/β) directly block RANK downstream signaling and inhibit osteoclast differentiation, while IFN-γ plays a context-dependent role that can either suppress or enhance RANKL expression [9]. Macrophages support osteoblast function and bone homeostasis under physiological conditions, whereas under inflammation they act as osteoclast precursors and produce osteoclastogenic cytokines such as TNF-α, IL-1, and IL-6 [36]. Because the IL-23/IL-17 axis is the major pathway in psoriasis, it could also contribute to skeletal abnormalities beyond joint involvement. Together, these mechanisms provide a biological rationale for the skin–bone axis, in which cutaneous inflammation may influence bone metabolism even without clinically apparent arthritis. These mechanisms have, however, been characterized principally in rheumatoid arthritis, ankylosing spondylitis and murine systems, so that their operation in human psoriasis is inferred rather than demonstrated (Level B, Table 1). The most direct experimental support comes from a murine model of chronic skin inflammation, in which epidermal inflammation caused systemic bone loss through IL-17-mediated inhibition of Wnt signalling in osteoblasts [20]. This remains the principal experimental demonstration that cutaneous inflammation alone can compromise the skeleton, and no equivalent mechanistic study has been performed on human psoriatic skin and bone.

## 4. Bone Abnormalities in Psoriasis Beyond Joint Involvement: Epidemiology

### 4.1. Prevalence of Osteoporosis and Low Bone Mineral Density

The reported prevalence of osteoporosis in psoriatic disease varies widely according to the population studied, ranging from 1.4% to 68.8% across studies [37]. For example, a cross-sectional study of 58 psoriasis patients not receiving systemic or biologic therapy demonstrated significantly lower hip and lumbar spine BMD in psoriasis patients than in healthy controls [13]. An Italian study of 43 patients with psoriatic disease, 19 of whom had PsA, found that those with longer disease duration had a higher prevalence of osteopenia and osteoporosis, suggesting a cumulative effect of chronic inflammation on bone homeostasis [14]. Beyond dual-energy X-ray absorptiometry (DXA), high-resolution peripheral quantitative computed tomography (HR-pQCT) has revealed cortical and trabecular microarchitectural abnormalities and reduced bone strength even in cutaneous psoriasis without arthritis [21]. This raises the possibility that BMD assessment by DXA alone may underestimate skeletal deficits in this patient group. These observations nevertheless require careful appraisal. The individual studies are small, single-centre and cross-sectional; they differ in the skeletal sites measured and in the control populations used; and they adjust inconsistently for body mass index, vitamin D status, smoking and prior corticosteroid exposure. Most did not exclude PsA by structured rheumatologic assessment. Screening questionnaires such as the Psoriasis Epidemiology Screening Tool [38] are useful for detecting clinically evident PsA but cannot reliably exclude subclinical enthesitis or synovitis; where these are present, the resulting bone phenotype cannot be attributed to cutaneous inflammation alone, and such attribution would require rheumatologic examination and, in research settings, imaging. Consistent with this heterogeneity, the largest meta-analysis to date, pooling 15 observational studies and more than 1.2 million participants, found no significant difference in areal BMD between patients with psoriatic disease and controls at the lumbar spine (standardized mean difference 0.07, 95% confidence interval (CI) −0.19 to 0.32), femoral neck (−0.08, −0.36 to 0.20) or total hip (−0.05, −0.22 to 0.13), with substantial between-study heterogeneity (I^2^ 34.7–73.8%), and no significant increase in the adjusted odds of osteoporosis (odds ratio 1.33, 95% CI 0.78–2.26; I^2^ 92.7%) [15]. A separate meta-analysis of HR-pQCT studies did report lower mean volumetric BMD in psoriatic disease, but its constituent studies pooled cutaneous psoriasis with PsA, and trial sequential analysis indicated that the accumulated sample size remained insufficient for the trabecular and cortical compartments [22]. A reduction in areal BMD in cutaneous psoriasis is therefore not established; microarchitectural abnormality is a more promising but still unconfirmed finding (Level B), and adequately powered studies in patients screened to exclude joint involvement are required.

### 4.2. Fracture Risk

Population-based studies provide complementary, and partly discordant, evidence regarding clinically meaningful skeletal compromise. In the meta-analysis cited above, the pooled adjusted odds of fracture were significantly but only slightly increased in psoriatic disease (odds ratio 1.09, 95% CI 1.06–1.12), with no detectable between-study heterogeneity (I^2^ 0%) [15]. This meta-analysis included the United Kingdom cohort discussed below [17], so that the two cannot be regarded as independent replications of one another. This pooled estimate derives from cohorts in which cutaneous psoriasis and PsA were combined—the same limitation we raise against the imaging meta-analyses—and cannot therefore be attributed to psoriasis without arthritis alone. The juxtaposition of this small fracture signal with the null pooled estimates for BMD and osteoporosis is the central observation of the epidemiologic literature: if it reflects a real biological effect, it implies a deficit in bone quality rather than in bone quantity. The same authors, however, reported divergence between unadjusted and adjusted estimates and interpreted this as evidence of substantial confounding [15], so an alternative explanation—that fractures in psoriasis are driven by treatment exposure, comorbidity and falls risk rather than by inflammation acting on bone—cannot be excluded on present data.

The two largest individual cohorts reach opposite conclusions, and the reasons are instructive rather than incidental. The Norwegian HUNT study integrated hospital-derived fracture data with information on psoriasis status, BMD measurements, lifestyle factors, National Education Database–based socioeconomic data, and prescription databases; among 48,194 participants including 2804 psoriasis patients, no significant increase in fracture risk was observed (age- and sex-adjusted hazard ratio (HR) 1.03, 95% CI 0.82–1.31) and no clear association between BMD T-score at total hip, femoral neck, or lumbar spine and psoriasis was found [16]. In contrast, a population-based cohort study using the United Kingdom Health Improvement Network (THIN) data from 1994 to 2014 compared PsA patients (*n* = 9788), psoriasis patients (*n* = 158,323), and controls (*n* = 821,834), and found that patients with mild psoriasis (HR 1.07, 95% CI 1.05–1.10) and severe psoriasis (HR 1.26, 95% CI 1.15–1.39) exhibited a severity-dependent increase in fracture risk [17]; the largest single effect was for vertebral fracture in severe disease (adjusted HR 2.23, 95% CI 1.54–3.22) [17]. In neither cohort was PsA excluded by systematic rheumatologic assessment.

Three methodological differences account for much of this discordance. First, outcome ascertainment differed: the HUNT analysis evaluated forearm and hip fractures only, whereas the THIN analysis captured fractures at all sites, and the strongest signal in THIN was vertebral—a site HUNT did not assess. Second, exposure ascertainment differed: psoriasis in HUNT was self-reported, and the resulting non-differential misclassification would be expected to bias the estimate toward the null, whereas THIN used physician-entered diagnostic codes. Third, HUNT did not stratify by disease severity; if the association is confined to severe disease, as the THIN data indicate, a predominantly mild, community-ascertained cohort will dilute it, and with 2804 cases HUNT had limited power to detect a hazard ratio of the magnitude reported for mild psoriasis. The THIN analysis is not free of bias in the opposite direction: severity was defined by receipt of phototherapy or systemic therapy, so the severe stratum is inseparable from exposure to systemic corticosteroids, ciclosporin, methotrexate and phototherapy; smoking, alcohol intake, body mass index and falls risk are captured incompletely in primary-care records; and patients under active specialist care undergo more imaging, which can inflate the recorded incidence of clinically silent vertebral fracture. On balance, the evidence supports at most a modest, severity-dependent association between psoriasis and fracture, observed in one large cohort [17] but not in another [16], and in a meta-analysis that pooled psoriasis with PsA and included the former cohort [15] (Level B as an association). This association remains vulnerable to residual confounding and should not yet be regarded as an established component of the skin–bone axis; the inference that cutaneous inflammation is itself the cause is hypothesis-generating (Level C), because no observational design published to date separates the effect of inflammation from that of its treatment, its comorbidities and the behaviours that accompany it. Resolving this will require cohorts in which psoriasis severity is defined independently of therapy, subclinical arthritis is excluded prospectively, and vertebral fracture is ascertained by systematic imaging rather than by clinical coding.

### 4.3. Circulating Bone Biomarkers

In addition to imaging findings, circulating biomarkers also support a systemic skin–bone interaction. Bone turnover markers can be broadly categorized into those reflecting osteoblast activity (i.e., bone formation), such as procollagen type I N-terminal propeptide (P1NP), bone-specific alkaline phosphatase, and osteocalcin, and those reflecting osteoclast activity (i.e., bone resorption), such as C-terminal telopeptide of type I collagen (CTX), N-terminal telopeptide of type I collagen (NTX), and tartrate-resistant acid phosphatase 5b. The latter quantifies fragments cleaved from bone matrix collagen during osteoclast-mediated resorption, while the former quantifies fragments generated during the synthesis and secretion of new collagen by osteoblasts. These markers reflect shorter-term dynamics of bone metabolism than BMD measurement and are also used to monitor therapeutic responses to anti-resorptive and bone-forming agents.

In a clinical study of human psoriasis patients in Poland, Bartosińska et al. reported significantly elevated serum levels of COMP, OPG, and IL-20 in a cohort of 61 patients with psoriasis, 22 of whom had PsA; OPG and IL-20 were elevated both in the 39 patients without arthritis and in those with PsA [12]. Results from studies measuring CTX and P1NP have been less consistent; however, in the study by Mentzel et al., which compared age-, sex-, and body mass index (BMI)-matched healthy controls with patients with psoriasis vulgaris (PsA status not reported), the bone formation marker P1NP was significantly reduced in the psoriasis group, while the bone resorption marker CTX did not differ from controls, and the reduced P1NP did not recover despite improvement in the Psoriasis Area and Severity Index (PASI) with anti-psoriatic therapy [23]. These findings suggest that psoriasis may be associated with impaired bone formation that did not normalize with improvement in skin disease, and raise the possibility that any skeletal imbalance in this disease involves “reduced bone formation” rather than “increased bone resorption.” This was, however, a single case–control study of modest size whose findings have not been independently replicated, and cross-sectional differences in turnover markers cannot establish the direction of the relationship; that psoriasis is characterized by suppressed bone formation rather than by accelerated resorption should therefore be regarded as a hypothesis worth testing rather than as an established feature (Level C).

The Wnt/β-catenin pathway is the central regulator of osteoblast differentiation and bone formation, governing lineage commitment from mesenchymal precursors to osteoblasts, Runx2- and Osterix-mediated maturation, and OPG production–mediated suppression of osteoclastogenesis, thereby maintaining the coupling of bone formation and resorption in a positive direction. Endogenous antagonists of this pathway, namely sclerostin (osteocyte-derived) and Dickkopf-1 (DKK-1), bind to the Wnt co-receptor LRP5/6 to block Wnt signaling, reduce osteoblast activity and OPG production, and consequently induce both suppression of bone formation and enhancement of bone resorption. In PsA and in experimental models, TNF-α and IL-17 can suppress the Wnt pathway by inducing these antagonists [11,20,35], and within the skin itself, Wnt5a and FZD2/5 are overexpressed in psoriatic lesional keratinocytes and participate in epidermal proliferation and architectural abnormalities [39], suggesting that the Wnt pathway is a common molecular substrate involved in the pathology of both skin and bone. In PsA, DKK-1 has attracted attention as a candidate molecule linking inflammatory cytokine signaling to suppressed bone formation and aberrant new bone formation [24]; however, studies directly measuring serum sclerostin/DKK-1 in psoriasis without arthritis remain limited and represent a future research priority.

### 4.4. Confounding by Lifestyle, Comorbidity, and Treatment Exposure

Any appraisal of the epidemiologic data must account for the fact that psoriasis is accompanied by a characteristic cluster of exposures, each of which independently affects the skeleton (Figure 1, lower box). Smoking, excess alcohol intake, physical inactivity, obesity and disturbed sleep are all over-represented in psoriasis and are themselves exacerbating factors for the skin disease [40]; smoking suppresses osteoblast function and promotes bone resorption, and habitual intake of more than two alcoholic drinks per day raises the relative risk of hip fracture to approximately 1.4 [41]. Vitamin D deficiency is common in psoriasis, reflecting both altered cutaneous vitamin D metabolism and reduced ultraviolet exposure [42], and impairs intestinal calcium absorption and bone mineralization. The comorbidities that cluster with psoriasis—inflammatory bowel disease, non-alcoholic fatty liver disease, chronic kidney disease, type 2 diabetes and depression—each carry an independent skeletal penalty, through malabsorption, impaired vitamin D activation, mineral and bone disorder, accumulation of advanced glycation end-products, and reduced physical activity [43]. None of the cohort studies discussed above adjusted for all of these simultaneously.

Treatment exposure is a particularly under-appreciated confounder. Systemic glucocorticoids are not standard therapy for psoriasis, but intermittent exposure occurs through the management of comorbidities, and potent and very potent topical corticosteroids—prescribed to most patients with moderate-to-severe disease—were associated with a dose-dependent increase in the risk of osteoporosis and major osteoporotic fracture in a Danish nationwide cohort of 723,251 adults treated with these agents [44]. The magnitude is material in the present context: the hazard ratio for major osteoporotic fracture rose from 1.05 at a cumulative dose of 1000–1999 g to 1.27 at 10,000 g or more [44], the latter of the same order as the hazard ratio of 1.26 reported for fracture in severe psoriasis [17]. Because cumulative topical corticosteroid exposure rises with psoriasis severity, it is collinear with the very severity gradient that is taken as evidence of an inflammation-driven effect on bone [17]. Disentangling the two will require designs that measure cumulative corticosteroid dose explicitly; until this is done, the severity–fracture gradient should not be read as direct evidence that cutaneous inflammation increases fracture risk.

## 5. The Bone Marrow Immune Niche in Psoriasis

The content of this section is, in its entirety, hypothesis-generating (Level C, Table 1). To our knowledge, no study has examined bone marrow tissue, hematopoietic progenitor phenotype, or niche stromal composition in patients with psoriasis. The mechanisms outlined below are inferred from murine models and from other chronic inflammatory states, and they are presented here because they define a testable research agenda, not because they are supported by direct evidence in this disease.

The bone marrow is a specialized immune microenvironment in which long-lived memory T cells, plasma cells, and innate lymphoid cells reside in close proximity to mesenchymal stromal cells (including CXCL12-abundant reticular (CAR) cells and Lepr^+^cells), osteoblasts, and endothelial cells, with stromal-derived CXCL12, IL-7, and stem cell factor maintaining hematopoietic stem cells and lymphocyte populations [45,46]. The bone marrow also serves as a reservoir of osteoclast precursors, which differentiate into mature osteoclasts in response to RANKL and macrophage colony-stimulating factor (M-CSF) [47]. Under chronic inflammatory conditions, inflammatory cytokines and chemokines promote osteoclast precursor expansion and differentiation, thereby linking immune activation to bone remodeling. The possibility that skin-tropic IL-17–producing memory T cells accumulate in the bone marrow of psoriasis patients has not been examined, and we raise it only as a hypothesis that the bone marrow might function as a long-term reservoir capable of sustaining or relapsing cutaneous inflammation.

Under chronic systemic inflammation—as characterized in models of infection, atherosclerosis and rheumatoid arthritis rather than in psoriasis—the bone marrow niche can itself undergo substantial remodeling. In these systems, persistent inflammatory cytokines, including TNF-α, IL-17, and IL-23, disrupt stromal cell function, alter CXCL12 gradients that regulate hematopoietic cell retention, impair osteoblast-mediated niche maintenance, and promote osteoclastogenic differentiation. These changes shift the bone marrow from a homeostatic microenvironment toward a pro-inflammatory niche that favors myelopoiesis, inflammatory monocyte production, and osteoclast precursor expansion. Whether this occurs in psoriasis is unknown; if it does, remodeling of the niche would provide a mechanistic link between persistent cutaneous inflammation and systemic skeletal abnormalities in psoriasis. A related concept is that hematopoietic stem and progenitor cells (HSPCs) can be epigenetically reprogrammed by systemic inflammatory signals to generate “trained” myeloid progeny that are more responsive to subsequent stimuli—a concept termed “central trained immunity” that has been demonstrated principally in models of infection and atherosclerosis [25,46]. It is plausible, but at present unsupported by any data in psoriasis, that sustained IL-23 and TNF-α signaling biases HSPC output toward inflammatory monocytes and osteoclast precursors. Testing these propositions in humans would require, at a minimum, comparison of circulating osteoclast precursor frequencies and of hematopoietic progenitor epigenetic profiles between patients with psoriasis and matched controls, ideally with paired assessment of bone microarchitecture; no such study has been reported.

## 6. The Microbiome–Bone–Psoriasis Axis

In a small study of patients with recent-onset PsA (*n* = 16), patients with skin psoriasis (*n* = 15) and healthy controls, gut microbial diversity was reduced in both patient groups, whereas reductions in *Akkermansia* and *Ruminococcus* were confined to PsA [26]. Among microbial metabolites, short-chain fatty acids (SCFAs; acetate, propionate, butyrate) increase intestinal and bone marrow Tregs, suppress osteoclast differentiation, and promote osteoblast-mediated bone formation, as established within bone metabolism research. Specifically, in an animal experiment in which *Lactobacillus rhamnosus* GG was orally administered, butyrate produced by the bacteria contributed to bone mass increase by Treg-mediated upregulation of osteoblast Wnt10b expression [27], suggesting that interventions that increase SCFAs may also benefit bone health. Two limitations of this inference deserve emphasis: the experiment was performed in mice without skin inflammation, using a probiotic strain that has not been shown to be depleted in psoriasis, and the dysbiosis reported in psoriatic disease derives from small cohorts in which the more pronounced taxonomic changes were found in PsA rather than in skin psoriasis [26]. No human study has related faecal or circulating short-chain fatty acid concentrations to bone mineral density, bone turnover, or fracture in psoriasis.

Aromatic indoles as tryptophan metabolites are produced by intestinal *Lactobacillus*, *Clostridium*, and *Bifidobacterium* species from tryptophan substrate and function as endogenous ligands of the aryl hydrocarbon receptor (AhR) [48]. AhR regulates intestinal and cutaneous barrier immunity through Th17/Treg balance and IL-22 production, and AhR activation attenuates psoriasiform inflammation in murine models and in human psoriatic skin [49,50]; consistent with this, the AhR agonist tapinarof has been approved as a topical therapy for psoriasis [51]. A reduction in AhR ligands has been reported in osteoporosis model mice, suggesting potential propagation to the bone–immune axis [52]. Although the possibility that reduced production of these protective metabolites in the dysbiotic psoriatic gut contributes to an inflammatory bone phenotype is theoretically plausible, direct evidence in psoriasis is currently lacking, and verification of the microbiome–bone axis remains an important future research priority. The AhR–bone data derive from receptor-deficient mice rather than from psoriasis [52]; no human study has linked tryptophan-derived AhR ligands to skeletal outcomes in this disease, and the microbiome–bone–psoriasis axis should therefore be regarded as Level C throughout. It is included here as a research priority rather than as a component of the current evidence base.

## 7. Biologic Therapy and Effects on Bone

Recent reviews have summarized the effects of targeted therapies on bone in psoriatic disease [53]. In interpreting these data, however, it is essential to recognize that the skeletal outcomes reported are biologically and clinically distinct and are not interchangeable: local joint erosion, pathological new bone formation such as syndesmophytes and enthesophytes, radiographic progression, systemic bone mineral density, and fracture. Suppression of erosive progression in PsA does not demonstrate preservation of systemic bone mineral density or a reduction in fracture risk, and reduced radiographic progression in ankylosing spondylitis is not equivalent to protection against osteoporosis. No adequately powered trial has demonstrated that any biologic agent reduces fracture risk in patients with cutaneous psoriasis, and the sections that follow therefore specify whether each observation derives from cutaneous psoriasis, PsA, or other inflammatory diseases.

### 7.1. Cytokine Pathway–Targeted Biologic Agents (TNF-α, IL-17, and IL-23 Inhibitors)

Biologic agents targeting TNF-α, IL-17, and IL-23 have sequentially transformed the treatment of psoriasis and PsA; however, their skeletal effects differ in mechanistic rationale and clinical evidence depending on the targeted pathway. TNF-α inhibitors were the first biologic class introduced for this disease group, and TNF-α blockade is expected to restore bone homeostasis by eliminating the principal drivers of RANKL induction, osteoblast suppression, and Wnt antagonism [11]. In ankylosing spondylitis, a meta-analysis showed significant improvement of BMD with anti-TNF therapy (mean increase of approximately +5–9% at the lumbar spine and +2–3% at the total hip, with femoral neck bone mineral density remaining stable) [28], and similar trends have been suggested in PsA, although characterization is more limited. These lumbar spine gains may, however, partly reflect syndesmophytes and other new bone formation captured by dual-energy X-ray absorptiometry rather than a true gain in bone mass, and none of these data address fracture. Direct evidence in cutaneous psoriasis without arthritis remains limited; small uncontrolled studies suggest favorable bone marker changes during TNF-α inhibitor therapy, but adequately powered prospective studies with hard skeletal endpoints are lacking. No randomized or prospective controlled study has evaluated bone mineral density, bone microarchitecture, or fracture as an endpoint in cutaneous psoriasis for any biologic agent. The expectations set out in this section are therefore extrapolations: Level B where they rest on data from ankylosing spondylitis, rheumatoid arthritis or PsA, and Level C where they rest on mechanism alone.

IL-17 pathway inhibitors might be expected to confer skeletal benefit given the central role of IL-17A in osteoclastogenesis, RANKL induction, and Wnt antagonism [18,20]. The clinical study in PsA showed that secukinumab resulted in the arrest of local catabolic and anabolic bone changes at the joint alongside resolution of synovitis [29]; that study was, however, small and uncontrolled, so it cannot distinguish a treatment effect on bone from the natural course of skeletal change following control of synovitis. In ankylosing spondylitis, IL-17A inhibition has been associated with limited radiographic progression, but reduced radiographic progression is not equivalent to preservation of systemic bone mass or protection against fracture. Direct trabecular BMD data in cutaneous psoriasis treated with IL-17 inhibitors remain scarce but represent a high-yield area for future investigation. Real-world cohorts now document sustained treatment persistence with IL-17 inhibition, as shown for bimekizumab in a multicentre Italian study with an estimated 2-year drug survival of 82.4% [54]; such cohorts could provide the long-term exposure needed to assess skeletal outcomes, which have not yet been reported.

Selective IL-23 inhibitors target the p19 subunit and attenuate Th17 differentiation upstream of IL-17 production. Preclinical data show that IL-23 overexpression is sufficient to induce systemic bone loss [55], providing a mechanistic rationale, derived from mice, for a skeletal effect of IL-23 blockade. However, bone-specific endpoints have rarely been reported in the major IL-23 inhibitor trials, which had skin and joint outcomes as primary endpoints. The first comparative data on fracture have come from a retrospective, propensity-matched cohort of adults with psoriasis in a United States electronic health record network, in which initiators of risankizumab had a lower 5-year risk of shoulder or upper arm fracture (risk ratio 0.49, 95% CI 0.34–0.72) and of lumbar or pelvic fracture (0.54, 0.37–0.79) than initiators of methotrexate, with no difference at 90 days or 2 years and no difference in femoral fracture [56]. This comparison is subject to confounding by indication, methotrexate is not a neutral comparator, and psoriasis was identified from health records without rheumatologic assessment; the finding is therefore hypothesis-generating rather than evidence of skeletal protection. As long-term registry data accumulate, head-to-head comparisons of IL-23 versus IL-17 inhibitors with respect to skeletal effects will become feasible and clinically informative.

### 7.2. JAK and TYK2 Inhibitors

Oral small-molecule inhibitors of the Janus kinase (JAK) and tyrosine kinase 2 (TYK2) family have expanded therapeutic options but raise unique bone-related considerations: JAK-STAT (signal transducer and activator of transcription) signaling is essential for multiple bone-relevant cytokines including IL-6, IL-23, and the interferons, and preclinical data are mixed. In arthritis models, tofacitinib has been shown to suppress bone destruction through RANKL production inhibition [57], and JAK inhibition has been shown to enhance osteoblast function under steady-state conditions and increase bone mass [58]. In contrast, in a post hoc analysis of the ORAL Surveillance trial [59], conducted in patients with rheumatoid arthritis aged 50 years or older with at least one cardiovascular risk factor, the incidence of all fractures was numerically higher with tofacitinib 5 mg and 10 mg twice daily than with TNF-α inhibitors (hazard ratio 1.23, 95% CI 0.96–1.58, and 1.26, 0.97–1.62, respectively), and the risk of osteoporotic fracture was significantly higher with tofacitinib 10 mg twice daily (1.60, 1.09–2.36; 5 mg twice daily, 1.35, 0.91–2.01) [60]. These analyses were exploratory, fractures were captured as adverse events rather than by systematic ascertainment, and the findings, which derive from rheumatoid arthritis, cannot be directly extrapolated to psoriasis; they nevertheless indicate that a skeletal benefit of JAK inhibition, suggested by preclinical data, cannot be assumed in patients. As use of JAK inhibitors expands, dermatology-specific studies with longer follow-up will be informative.

Evidence specific to TYK2 inhibition is more limited. In the phase 3 POETYK PsA-1 trial in biologic-naive patients with PsA and radiographic erosions, deucravacitinib improved clinical outcomes compared with placebo, and a post hoc analysis indicated less progression of structural joint damage at week 16, sustained to week 52 [61]. This is a structural joint outcome in PsA, however, and no data are yet available on bone mineral density, bone turnover markers, or fracture risk with TYK2 inhibition in either PsA or cutaneous psoriasis.

### 7.3. Apremilast (PDE4 Inhibitor)

Apremilast is an oral phosphodiesterase 4 (PDE4) inhibitor that elevates intracellular cyclic adenosine monophosphate (cAMP), thereby suppressing the production of inflammatory cytokines including TNF-α, IFN-γ, IL-2, IL-12, IL-17, and IL-23, while concurrently enhancing anti-inflammatory mediators such as IL-10 [62]. As these cytokines regulate bone remodeling in other inflammatory settings, PDE4 inhibition could in principle influence bone homeostasis, although the direction of any effect is uncertain. However, the direct effect of PDE4 inhibition on bone is complex: preclinical studies have shown that PDE4 inhibition restores cAMP levels in osteoclasts and ameliorates osteoclast dysfunction in autosomal dominant osteopetrosis type 2 (ADO2) models, while in osteoblasts, elevated cAMP can upregulate TRANCE/RANKL expression [63]. Direct studies on the effects of apremilast on BMD or fracture risk in psoriatic disease are currently limited. Whether apremilast affects skeletal outcomes in psoriasis is therefore an open research question rather than a basis for treatment selection. Prospective evaluation of the effects of apremilast on bone turnover markers, BMD, and fracture endpoints in psoriasis cohorts is needed.

Taken together, the available evidence does not support selecting a TNF-α, IL-17, IL-23, TYK2, JAK, or PDE4 inhibitor specifically for skeletal protection in cutaneous psoriasis.

## 8. Conclusions

Psoriasis has long been regarded as a chronic inflammatory skin disease with systemic comorbidities, while skeletal abnormalities have generally been considered a secondary consequence of PsA. However, the accumulating evidence summarized in this review challenges this traditional view. Clinical studies have reported that patients with psoriasis without clinically apparent arthritis may exhibit altered bone microarchitecture, circulating abnormalities in bone turnover biomarkers, and a modestly increased risk of fracture, although much of this evidence derives from populations in which PsA was not systematically excluded. Although individual studies differ in design and magnitude of effect, these findings collectively suggest that skeletal involvement may represent an underrecognized systemic manifestation of psoriasis rather than merely a complication of joint disease. It is useful to state precisely what this amounts to. What the data support is narrow: one large cohort reported a modest, severity-dependent excess risk of fracture in psoriasis [17] that was not observed in a second large cohort [16], and a meta-analysis that pooled psoriasis with PsA and included the former cohort reported a small pooled association [15], which is not accompanied by a demonstrable reduction in areal bone mineral density [15]. What remains unresolved is whether this discrepancy reflects a genuine inflammation-driven deficit in bone quality or residual confounding by treatment exposure, comorbidity and lifestyle. What is at present speculative—bone marrow niche remodeling, central trained immunity, and microbiome-mediated skeletal regulation—has no direct supporting data in human psoriasis at all, and is offered here as a research agenda rather than as an explanation.

The concept of the skin–bone axis in psoriasis provides a biologically plausible framework that integrates these observations. Chronic cutaneous inflammation driven by the IL-23/IL-17 pathway may extend beyond the skin through immune-mediated mechanisms that, in other inflammatory diseases and experimental models, influence osteoclast differentiation, osteoblast function, and bone remodeling. Osteoimmunological pathways centered on the RANKL–RANK–OPG axis provide the molecular basis for this interaction, while it is conceivable, although unsupported by direct data in psoriasis, that inflammatory remodeling of the bone marrow niche could further amplify systemic immune activation through altered hematopoiesis, expansion of osteoclast precursors, and trained immunity. In parallel, gut microbial dysbiosis and reduced production of protective metabolites, including short-chain fatty acids and aryl hydrocarbon receptor ligands, could, in principle, modulate immune homeostasis and skeletal metabolism. If confirmed, these mechanisms might represent interconnected components of a systemic immune network linking persistent skin inflammation to bone homeostasis.

Importantly, many aspects of this framework remain hypothetical. Direct evidence demonstrating inflammatory remodeling of the bone marrow niche in psoriasis, migration or persistence of pathogenic memory T cells within bone marrow, and causal links between microbiome-derived metabolites and skeletal abnormalities in human psoriasis are currently lacking. Future investigations integrating longitudinal clinical cohorts, high-resolution skeletal imaging, multi-omics approaches, single-cell transcriptomics, spatial transcriptomics, and experimental animal models will be required to establish these mechanistic relationships. Furthermore, prospective studies should determine whether changes in bone biomarkers, bone mineral density, or bone microarchitecture correlate with disease activity, cumulative inflammatory burden, and therapeutic response. Such studies will clarify whether skeletal abnormalities represent reversible inflammatory manifestations or permanent structural consequences of chronic disease.

From a clinical perspective, recognition of the skin–bone axis has several important implications. Bone health should not be considered solely in patients with established PsA but may warrant attention in selected patients with cutaneous psoriasis, particularly those with severe disease, long disease duration, recurrent systemic inflammation, or additional osteoporosis risk factors. Advances in imaging modalities such as high-resolution peripheral quantitative computed tomography and the identification of sensitive circulating bone biomarkers may facilitate earlier recognition of skeletal involvement before clinically overt fractures occur. Moreover, whether biologic therapies targeting TNF-α, IL-17, or IL-23 can preserve bone quality in addition to improving skin disease represents an important unanswered question with potential implications for treatment selection. At present, however, no evidence supports selecting any class of systemic therapy specifically for skeletal protection in cutaneous psoriasis. Equally, psoriasis alone is not currently an indication for routine assessment of bone mineral density by DXA, of bone microarchitecture by HR-pQCT, or of bone turnover markers. Skeletal assessment in patients with psoriasis should follow general osteoporosis-screening guidelines and be guided by individualized evaluation of established fracture risk factors, such as age, sex, prior fracture, glucocorticoid exposure, low body weight and family history of fracture.

This review has limitations. It is narrative rather than systematic: the literature search, although structured around predefined terms (Supplementary Table S1), was restricted to a single database and to English-language publications and was not exhaustive, so that relevant studies may have been missed. Although the grading scheme summarized in Table 1 was applied consistently, it is an operational construct rather than a validated instrument, and the assignment of levels necessarily involved judgement. Literature screening and the assignment of evidence levels were performed by a single reviewer without independent duplication, which increases the risk of selection bias and of subjective grading in a literature as heterogeneous as this one. The clinical literature is dominated by cross-sectional studies of modest size in which subclinical psoriatic arthritis was rarely excluded, so the distinction between psoriasis without arthritis and early psoriatic arthritis—on which the central claim of this review depends—is less secure in the source data than the terminology implies. Screening questionnaires do not resolve this, because they cannot exclude subclinical enthesitis or synovitis. Publication bias is likely to favour positive skeletal findings in this field. Finally, the mechanistic sections draw on models of skin inflammation and on systemic inflammation in other diseases; the internal coherence of the resulting narrative should not be mistaken for evidence that the pathway operates in patients.

Ultimately, the skin–bone axis should not be viewed as a replacement for existing concepts of psoriasis as a systemic inflammatory disease, but rather as an extension of them. By integrating osteoimmunology, bone marrow biology, microbiome research, and clinical skeletal observations into a unified conceptual model, this framework provides a new perspective for understanding the systemic consequences of psoriasis. Continued interdisciplinary research involving dermatology, immunology, bone biology, and microbiome science will be essential to validate this concept and to determine whether preservation of skeletal health should become an integral component of comprehensive psoriasis management in the future.

## Figures and Tables

**Table 1:** Strength of the evidence supporting each component of the proposed skin–bone axis in psoriasis. Levels are defined in Section 1: A, established in psoriatic disease; B, extrapolated from other inflammatory diseases, animal models, or general bone biology; C, hypothetical, with no direct supporting data in psoriasis. Population categories are defined in Section 1: i, cutaneous psoriasis with PsA prospectively excluded; ii, cutaneous psoriasis without systematic exclusion of PsA; iii, mixed psoriasis/PsA populations; iv, PsA alone; v, non-psoriatic inflammatory diseases or experimental models. BMD, bone mineral density; DKK-1, Dickkopf-1; HR-pQCT, high-resolution peripheral quantitative computed tomography; IL, interleukin; OPG, osteoprotegerin; P1NP, procollagen type I N-terminal propeptide; PsA, psoriatic arthritis; RANKL, receptor activator of nuclear factor-κB ligand; SCFA, short-chain fatty acid; TNF-α, tumor necrosis factor-α.

Component of the Proposed Axis	Principal Supporting Evidence	Population	Level	Principal Limitation
TNF-α/RANKL-driven osteoclastogenesis and bone erosion in PsA	Human and experimental mechanistic studies [11]	iv [11]	A (in PsA)	Not transferable to psoriasis without arthritis
RANKL–RANK–OPG axis; IL-17-driven osteoclastogenesis	Core osteoimmunology [8,9,18,19]	v	A (as bone biology); B (in psoriasis)	Characterized mainly in rheumatoid arthritis, ankylosing spondylitis and murine systems
Skin inflammation causing systemic bone loss via IL-17-mediated inhibition of osteoblast Wnt signalling	Murine model of chronic skin inflammation [20]	v [20]	B	Single model system; not demonstrated in human psoriasis
Reduced areal BMD in cutaneous psoriasis	Small cross-sectional studies [13,14]; meta-analysis null at all sites [15]	ii [13]; iii [14,15]	B	Pooled standardized mean differences non-significant (I^2^ 34.7–73.8%), so a reduction in areal BMD is not established; subclinical PsA rarely excluded; one study included patients with PsA [14]
Abnormal bone microarchitecture in psoriasis without arthritis	Single-centre HR-pQCT study [21]; HR-pQCT meta-analysis [22]	ii [21]; iii [22]	B	Small single-centre samples; the meta-analysis pooled psoriasis with PsA and its trial sequential analysis was inconclusive for the cortical and trabecular compartments
Increased fracture risk	Two discordant large cohorts [16,17]; pooled adjusted odds ratio 1.09 (1.06–1.12), I^2^ 0% [15]	ii [16,17]; iii [15]	B (as an association); C (as causation)	Treatment-based severity proxy; residual confounding; discordant outcome ascertainment; the meta-analysis pooled psoriasis with PsA and included cohort [17]
Suppressed bone formation (low P1NP) as the dominant abnormality	Single case–control study [23]	ii [23]	C	Not replicated; cross-sectional; direction of effect unknown
Sclerostin/DKK-1 involvement	Data in PsA only [24]	iv [24]	C (in cutaneous psoriasis)	Not measured in psoriasis without arthritis
Bone marrow niche remodelling in psoriasis	No human psoriasis data; inferred from other inflammatory states	v	C	Entirely hypothetical
Bone marrow as a reservoir of skin-tropic IL-17-producing memory T cells	No data in psoriasis	No data	C	Entirely hypothetical
Central trained immunity of hematopoietic progenitors	Concept established in infection and atherosclerosis models [25]	v [25]	C (in psoriasis)	Not demonstrated in psoriasis
Microbiome-derived metabolites regulating the skeleton	Reduced diversity in small psoriasis and PsA groups [26]; SCFA–bone link in mice [27]	ii and iv, analysed separately [26]; v [27]	C	No study relates microbial metabolites to skeletal outcomes in psoriasis
Skeletal benefit of biologic therapy	Extrapolated from ankylosing spondylitis and rheumatoid arthritis [28]; small PsA study [29]	v [28]; iv [29]	B (structural joint outcomes in PsA); C (BMD or fracture in cutaneous psoriasis)	No trial with BMD or fracture endpoints in cutaneous psoriasis; joint erosion, radiographic progression, BMD and fracture are distinct outcomes

## Data Availability

No new data were created or analyzed in this study. Data sharing is not applicable to this article.

## Supplementary Materials

The following supporting information can be downloaded at: https://www.mdpi.com/article/10.3390/jcm15208066/s1, Table S1: Literature search strategy.
