# Supplementary material for: The Skin-Bone Axis in Psoriasis: An Emerging Osteoimmunological Perspective
Source: J Clin Med. 2026 Oct 17;15(20):8066. doi: 10.3390/jcm15208066 (PMC13706361; doi:10.3390/jcm15208066)
Supplement: Supplementary file 1 [file jcm-15-08066-s001.zip › jcm-4479974-supplementary.pdf]

Supplementary Materials

The Skin-Bone Axis in Psoriasis: An Emerging Osteoimmunological Perspective

Natsuko Saito-Sasaki and Yu Sawada

Table S1. Literature search strategy.

Item	Description
**Review type**	Narrative review; the literature search was structured but not systematic
**Database searched**	PubMed (MEDLINE)
**Search period**	Database inception to March 2026; targeted searches for recently published studies were repeated during manuscript revision (September 2026)
**Language**	English-language publications only
**Publication type**	No restriction by publication type
**Search approach**	Iterative keyword searches combining disease terms with bone/mechanism terms (see below), supplemented by manual screening of the reference lists of retrieved articles. A single predefined search string was not used.
**Disease terms**	psoriasis; psoriatic arthritis; psoriatic disease
**Bone terms**	bone; bone mineral density; osteoporosis; osteopenia; fracture; bone metabolism; bone turnover; osteoclast; osteoblast; RANKL
**Mechanism terms**	osteoimmunology; IL-17; IL-23; TNF; Th17; bone marrow niche; trained immunity; microbiome; short-chain fatty acid; aryl hydrocarbon receptor
**Representative combinations**	psoriasis AND bone; psoriasis AND osteoporosis; psoriasis AND fracture; psoriasis AND bone mineral density; psoriatic arthritis AND osteoporosis; psoriasis AND osteoimmunology; IL-17 AND osteoclast; psoriasis AND bone marrow; psoriasis AND microbiome AND bone
**Screening and evidence assignment**	Title/abstract screening, full-text assessment and assignment of evidence levels (Table 1) were performed by a single reviewer (N.S.-S.); dual independent screening was not undertaken

IL, interleukin; RANKL, receptor activator of nuclear factor-κB ligand; Th17, T helper 17; TNF, tumor necrosis factor.
